# Comparison of the efficacy and safety of sedation protocols with the use of dexmedetomidine–remifentanil and propofol–remifentanil during percutaneous closure of atrial septal defects: a randomized clinical trial

**DOI:** 10.1186/s13019-022-01834-6

**Published:** 2022-05-03

**Authors:** Xiao-Lan Chen, Wen-Hui Huang, Yi-Han Zheng, Gui-Can Zhang

**Affiliations:** 1 Department of Intensive Care Unit, the First Affiliate Hospital, Fujian Medical University, Fuzhou, 350004 Fujian Province People’s Republic of China; 2 Anesthesiology Research Institute, the First Affiliated Hospital, Fujian Medical University, Fuzhou, 350004 Fujian Province People’s Republic of China; 3 Department of Cardiovascular Surgery, Fujian Medical University Union Hospital, Fuzhou, 350001 Fujian Province People’s Republic of China

**Keywords:** Atrial septal defects, Sedation, Dexmedetomidine, Propofol, Remifentanil

## Abstract

**Background:**

The study was aimed to compare the efficacy and safety of different sedation protocols of dexmedetomidine–remifentanil and propofol–remifentanil for percutaneous closure of atrial septal defects (ASD) under transthoracic echocardiography (TTE) guidance.

**Material and methods:**

From March 2020 to January 2021, of 114 patients screened, 59 ASD patients scheduled for percutaneous closure under TTE guidance were randomly allocated into the dexmedetomidine–remifentanil (D–R) group (n = 29) and the propofol–remifentanil (P–R) group (n = 30). The incidence of hemodynamic and respiratory adverse events, arterial blood gas analysis, induction and recovery time, pain score, infusion rate of remifentanil, satisfaction of the surgeon and patient, additional sedatives were collected for analysis and comparison.

**Results:**

The induction time was longer in the D–R group than that in the P–R group (17.66 ± 2.65 min vs 11.43 ± 1.48 min; difference, 6.22 min; 95% CI 5.10 to 7.35; *P* < 0.001). No differences were observed in the 2 groups in terms of the additional sedatives, infusion rate of remifentanil, pain score, recovery time (*P* > 0.05). There was no difference between the two groups regarding the incidence of cardiovascular adverse events (6 [20.7%] vs 4 [13.3%]; difference, 7.4%; 95% CI − 11.7 to 26.5%; *P* = 0.506). Respiratory adverse events occurred in 1 patient (3.4%) in the D–R group, and 8 patients (26.7%) in the P–R group (difference, 23.3%; 95% CI 6.2 to 40.5%; *P* = 0.026). The incidence of hypercapnia was significantly lower in the D–R group (4 [13.8%]) than in the P–R group (13 [43.3%]; difference, 29.5%; 95% CI 7.8 to 51.2%; *P* = 0.012).

**Conclusions:**

Except for more rapid the induction time and higher the surgeon satisfaction score in the propofol–remifentanil protocol, the efficacy was similar between two sedation protocols. The hemodynamic stability was comparable, the dexmedetomidine–remifentanil protocol had superior airway security due to fewer hypercapnia and respiratory adverse events.

## Background

Percutaneous closure of atrial septal defect (ASD) has achieved satisfactory outcomes given the lower morbidity rates, superior cosmetic results and lower levels of postoperative pain compared to surgery [1]. Total transthoracic echocardiography (TTE), which is used to monitor and guide the procedure of percutaneous ASD closure, is a suitable alternative method for avoiding exposure from X-ray radiation and esophageal probe [2, 3]. The spontaneous movements and agitation during the surgery might induce technical difficulties and failures of the procedure. While proper sedation and analgesia can alleviate discomfort and provide amnesia.

Propofol has a rapid onset of action, and there is a fast recovery of cognition once propofol is discontinued. However, it is difficult to control sedation depth with propofol. And this sedative does not possess analgesic effects. In addition to dose-dependent respiratory depression, propofol might not retain airway security especially given in combination with opioid agonists in non-intubated patients [4, 5]. Thus, it is necessary for clinicians to seek a more appropriate drug of optimally retaining airway security during sedation. Dexmedetomidine is a selective and specific ɑ^2^-adrenoceptor agonist with easily roused sedation and analgesic properties. Dexmedetomidine does not decrease respiratory function when administered at appropriate doses. However, hemodynamic side effects associated with dexmedetomidine, including hypertension, hypotension and bradycardia, may limit its clinical application [6–9].

ASD patients may experience prolonged onset time and delayed peak time of intravenous drugs due to the presence of a left-to-right shunt. It will lead to undesirable respiratory and cardiovascular depression if delivery of sedatives is not carefully titrated [10, 11]. The procedure under TTE guidance did not require the similar sedation depth as transesophageal echocardiography, however, the shallower but equally effective sedation protocols with fewer sedative-related adverse events for this specific cohort of patients was imperative.

In this study, remifentanil combined with dexmedetomidine or propofol were all administered as a continuous intravenous infusion for achieving a proper sedation level. We hypothesized that there were more hemodynamic changes while fewer respiratory adverse events in the dexmedetomidine–remifentanil protocol in comparison to the propofol–remifentanil protocol.

## Materials and methods

This prospective, randomized and double-blind clinical research was approved by the Ethics Committee of Fujian Medical University, China (No. 2020KY018). Informed consent was obtained from all patients before the procedure. The study was registered at http://www.chictr.org.cn (No. ChiCTR2000030969).

### Patients

The inclusion criteria were ASD patients aged ≥ 18 years old, American Society of Anesthesiologists (ASA) physical status < IV and scheduled for percutaneous ASD device (Amplatzer atrial septal occluder) closure under TTE guidance. Indications for the procedure included hemodynamically significant left to right shunts, a single secundum ASD without any other intracardiac structural abnormality, sufficient rims, satisfactory transthoracic acoustic windows. The exclusion criteria were as follows: STOP-BANG score ≥ 3 (an 8-point score in which values higher than 3 are associated with intermediate risk of Obstructive Sleep Apnea), serious renal dysfunction (undergoing dialysis before surgery), serious hepatic dysfunction (Child–Pugh class C or D), serious heart dysfunction (left ventricular ejection fraction less than 30%), bradycardia (heart rate < 60 beats per minute or second- or third-degree block without pacemaker), known drug allergies, or a history of drug abuse [12, 13].

**Fig. 1:**
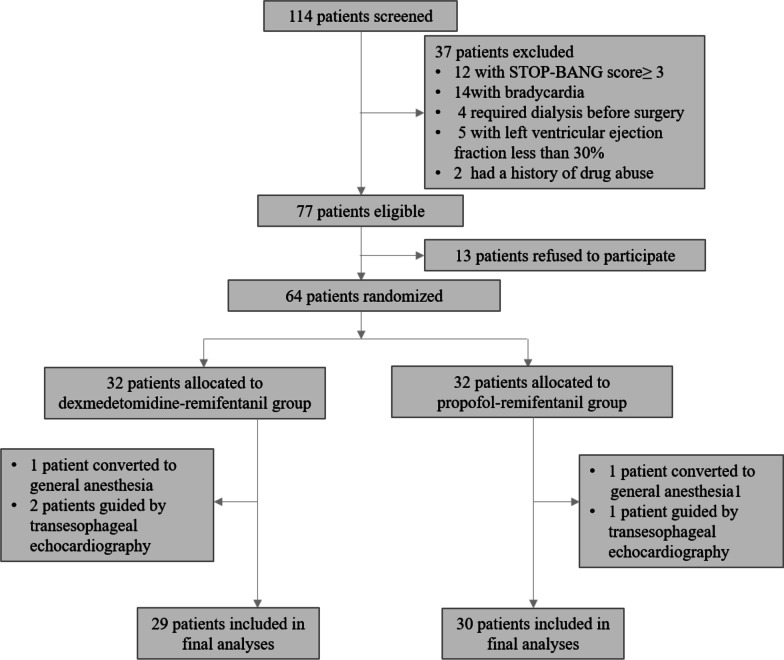
Participant flow diagram

From March 2020 to January 2021,114 patients were screened for study participation; of these, 64 patients were randomly allocated into the dexmedetomidine–remifentanil (D–R) group (n = 32) or the propofol–remifentanil (P–R) group (n = 32) using a random number table provided by www.random.org. Among the 64 patients, 2 patients (1 patient in the D–R group and 1 patient in the P–R group) were converted to surgical repair under general anesthesia and 3 patients (2 patients in the D–R group and 1 patient in the P–R group) were guided by transesophageal echocardiography (TEE) because of unsatisfactory transthoracic acoustic windows for evaluating postoperative residual shunt. Finally, 5 patients were excluded from the study, data for 59 patients (29 patients in the D–R group and 30 patients in the P–R group) were analyzed (Fig. 1).

### Blinding

All preoperative and intraoperative data were collected by a blind anesthesiologist not directly involved in sedation protocol. However, it is unrealistic for an attending anesthesiologist to be blind to preoperative and intraoperative data, which is critical to anesthesia management. Patients and surgeons were blinded to group allocation. All drug infusion pumps and infusion lines are concealed to avoid detection.

### Sedation protocols

None of the patients were premedicated. The sedation level was assessed with the Observer’s Assessment of Alertness/Sedation (OAA/S) scale (a ‘wide awake’ score = 5 and a ‘deeply sedated’ score = 1; the final score is the sum of the responsiveness, speech, facial expression, and eyes component scores) [14] and bispectral index (BIS, Aspect Medical System, Newton, MA, USA). Intraoperative sedation levels were targeted to achieve a BIS of 60–85 and an OAA/S score ≤ 4. Remifentanil (Ultiva®, China National Pharmaceutical Industry Corporation Ltd., HeBei, China) was infused continuously at a rate of 4 μg/kg per hour in both groups before starting the procedure. For the D–R group, the initial infusion of dexmedetomidine (Precedex®, Yangtze River Pharmaceutical (Group) Co. Ltd., JiangSu, China) was set at 6.0 μg/kg/h for 10 min as the initial loading dose, followed by a maintenance infusion beginning at a rate of 1.0 μg/kg/h. In the P–R group, the initial infusion of propofol (Pofol®, B.Braun Melsungen AG, Melsungen, Germany) was set at 6.0 mg/kg/h for 10 min, followed by continuous infusion beginning at a rate of 2 mg/kg/h. When target sedation level was obtained, the maintenance infusion rate of sedatives was adjusted according to the patients’ sedation level and all drugs were discontinued at the end of the procedure in both groups.

If sedation level was inadequate in either group, the infusion rates of sedatives were increased at first. Besides, a bolus of 10–20 mg propofol was administered as a rescue sedation therapy when first-line treatment failed [15].

### Anesthesia management and data collection

Vital signs were monitored continuously and recorded at 5-min intervals: oxygen saturation (SpO_2_), heart rate (HR), electrocardiogram (ECG), respiratory rate (RR). In addition, an arterial catheter was routinely inserted for assessing invasive arterial pressure, and arterial blood gas analysis was performed at baseline (breathing room air). All patients were breathing spontaneously, and 4 L/min oxygen was given through a nasal cannula. The sedation protocols were started after placement of the arterial catheter in both groups. On achieving the targeted sedation level (BIS of 60–85 and an OAA/S score ≤ 4), the right groin was infiltrated with 10 mL of 1% lidocaine at the beginning of the interventional procedure.

Arterial blood gas analysis was repeated immediately after the procedure. The number of patients with hypercapnia (PaCO_2_ ≥ 45 mmHg) was evaluated. The time needed to achieve an Aldrete score ≥ 9 (a10-point score in which values higher than 9 are adopted as the suggested criteria for discharge from the PACU; the final score is the sum of the activity, respiration, circulation, consciousness and O_2_ saturation component scores) was noted [16]. Patients were asked to evaluate their levels of pain (0 = no; 1–3 = mild; 4–6 = moderate; 7–10 = severe pain) by using the visual analogue scale (VAS) and were transferred to the ward when the Aldrete score was ≥ 9 [10]. The satisfaction with the quality of the sedation (5-point Likert scale: 1, very satisfied; 2, satisfied; 3, neutral; 4, dissatisfied; and 5, very dissatisfied) were evaluated by the surgeons and patients [13].

Hemodynamic and respiratory adverse events were defined as follows. Hypotension (mean arterial blood pressure < 65 mmHg), hypertension (mean arterial blood pressure ≥ 20% of baseline), bradycardia (heart rate < 50 beats per minute), bradypnea (respiratory rate < 8/min for ≥ 1 min), apnea (absence of ventilator effort ≥ 20 s) and oxygen desaturation (SpO2 < 90% for ≥ 10 s) were all recorded [13].

We managed adverse respiratory events with a jaw thrust, mask ventilation,

by increasing oxygen flow, or asopharyngeal/ oropharyngeal airway insertion. Noradrenaline, urapidil, atropine, or esmolol was administered for adverse hemodynamic events.

The efficacy of sedation protocols was assessed on the ability to successfully complete the procedure without rescue sedatives, pain score, infusion rate of remifentanil, induction time (the time to achieve targeted levels of sedation), recovery time (the time to an Aldrete score ≥ 9), anesthetic satisfaction of the surgeons and patients. The incidence of hemodynamic and respiratory adverse events, arterial blood gas analysis, hypercapnia all were compared to evaluate the safety of sedation protocols.

### Interventional procedure and echocardiography guidance

The patient was placed in a supine position, and TTE was performed continuously throughout the procedure to monitor device deployment. The interventional procedure of percutaneous closure of ASD has been described in the previous study [2]. Briefly, a venous sheath was inserted after the right femoral vein was punctured and systemic heparinization (1 mg/kg) was performed. Subsequently, a catheter was inserted, followed by advancement of a guidewire through the ASD into the left atrium. Then, an occluder was delivered carefully through the sheath. The left disc was deployed in the left atrium and pulled back against the atrial septum, and then the right atrial disc was deployed. Finally, the device was released once the occluder was positioned properly.

If patients did not undergo successful device implantation due to severe residual shunt, complete heart block, or device dislodged, then the patients were referred for surgical repair under general anesthesia with endotracheal intubation as a remedial measure and were excluded from the final sedation analysis.

### Statistical analysis

Statistical analyses were performed by SPSS software (ver.22.0, SPSS Inc., Chicago, IL, United States). The results are presented as the mean ± standard deviation (SD) for the continuous variables that were normally distributed or approximately normal distribution after normality test and histogram. The results are presented as the median and interquartile range (IQR) for those that were nonnormally distributed. Categorical variables are presented as numbers or percentages. Student’s t test and Mann–Whitney U-test were used to compare continuous variables with a normal distribution and those with a nonnormal distribution. For comparison of the categorical data, χ^2^ test or Fisher’s exact test was performed. Besides, for comparison of the grade data, Wilcoxon rank- sum test was performed. Effects are reported with a point estimate and 95% CI in addition to *P* values. All statistical tests were two-tailed with a significance level of 0.05.

The main safety concern with sedation in patients is respiratory adverse events. According to a previous study, propofol–remifentanil combinations for sedation during hysteroscopy, the incidence of respiratory depression was 40%. Based on this result, the decreases in the incidence of respiratory depression to 10% in dexmedetomidine–remifentanil group was considered clinically significant [17]. We estimated that 29 participants per group were needed considering a two-sided test with α = 0.05, power of 80% (β = 0.20). To compensate for possible loss, we assumed a 10% drop-out rate, and 3 participants were added to each group. Finally, a sample size of 32 subjects per group was required (total of 64 subjects) in this study.

## Results

The demographic and clinical characteristics are presented in Table 1. There were no significant differences between the two groups.

**Table 1:** The data of demographic and clinical characteristics

	D–R group (n = 29)	P–R group (n = 30)
Age (y)	45.34 ± 15.80	43.53 ± 16.31
Women, n (%)	20 (69.0%)	24 (80.0%)
Height (cm)	162.03 ± 8.34	160.03 ± 8.05
Weight (kg)	57.02 ± 10.06	57.88 ± 11.06
BMI (kg/m^2^)	21.62 ± 2.91	22.37 ± 3.19
ASA status (II/III), n	22/7	24/6
Snoring history, n (%)	8 (27.6%)	10 (33.3%)
Smoking history, n (%)	7 (24.1%)	5 (16.7%)
ASD size (mm)	18.96 ± 7.51	17.77 ± 6.88
PASP (mm Hg)	41.48 ± 9.04	39.03 ± 16.31
Qp/Qs	2.27 ± 0.32	2.18 ± 0.26

D–R group: dexmedetomidine–remifentanil group; P–R group: propofol–remifentanil group; BMI: body mass index; ASA: American Society of Anesthesiologists; ASD: atrial septal defect; PASP: pulmonary artery systolic pressure; Qp/Qs: pulmonary to systemic blood flow ratio

In terms of sedation efficacy (Table 2), all patients successfully completed procedure without apparent body movement leading to interruption of procedure. The induction time was longer in the D–R group than that in the P–R group (17.66 ± 2.65 min vs 11.43 ± 1.48 min; difference, 6.22 min; 95% CI 5.10 to 7.35; *P* < 0.001). Four patients (13.8%) in the D–R group and 2 patients (6.7%) in the P–R group required additional propofol administrated as a rescue sedative (difference, 7.1%; 95% CI − 8.3 to 22.5%; *P* = 0.424) at doses of 17.50 ± 5.00 mg and 15.00 ± 7.07 mg (difference, 2.50 mg; 95% CI − 10.94 to 15.94; *P* = 0.633), respectively. The infusion rates of remifentanil and maximal pain scores (VAS) were not significantly different in the two groups (*P* > 0.05). No difference between the D–R group and the P–R group was observed regarding the recovery time (13.03 ± 1.82 min vs 12.20 ± 2.17 min; difference, 0.83 min; 95% CI − 0.21 to 1.88; *P* = 0.116). All patients were transferred to the ward within 20 min after surgery. The patient satisfaction score was comparable (*P* = 0.668), whereas the surgeon satisfaction score was higher in P–R group than in D–R group (*P* = 0.006).

**Table 2:** Efficacy of sedation protocols in both groups

	D–R group (n = 29)	P–R group (n = 30)	Difference (95% CI)	*P* value
Induction time (min)	17.66 ± 2.65	11.43 ± 1.48	6.22 (5.10 to 7.35)	< 0.001
Procedure duration (min)	52.48 ± 9.11	52.23 ± 7.41	0.25 (− 4.07 to 4.57)	0.908
Sedation duration (min)	70.14 ± 9.62	63.67 ± 7.02	6.47 (2.09 to 10.85)	0.004
Remifentanil infusion rate (μg/kg/h)	3.99 ± 0.08	3.97 ± 0.09	0.02 (− 0.03 to 0.06)	0.481
Maximum pain level (VAS)			–	0.786
0 (no pain)	24 (82.8%)	24 (80.0%)		
< 3 (mild pain)	5 (17.2%)	6 (20.0%)		
Recovery time (min)	13.03 ± 1.82	12.20 ± 2.17	0.83 (− 0.21 to 1.88)	0.116
Additional propofol required				
Patients, n (%)	4 (13.8%)	2 (6.7%)	7.1% (− 8.3 to 22.5%)	0.424
Dose (mg)	17.50 ± 5.00	15.00 ± 7.07	2.50 (− 10.94 to 15.94)	0.633
Surgeon satisfaction score			–	0.006
1 (very satisfied)	11 (37.9%)	22 (73.3%)		
2 (satisfied)	12 (41.4%)	6 (20.0%)		
3 (neutral)	6 (20.7%)	2 (6.7%)		
Patient satisfaction score			–	0.668
1 (very satisfied)	23 (79.3%)	22 (73.3%)		
2 (satisfied)	4 (13.8%)	7 (23.3%)		
3 (neutral)	2 (6.9%)	1 (3.3%)		

D–R group: dexmedetomidine–remifentanil group; P–R group: propofol–remifentanil group; VAS: visual analog scale

Table 3 shows the incidence of intraoperative adverse events in both groups. In terms of sedation safety, there was no difference between the two groups regarding the incidence of cardiovascular adverse events (6 [20.7%] vs 4 [13.3%]; difference, 7.4%; 95% CI − 11.7 to 26.5%; *P* = 0.506). Two patients in D–R group developed transient hypertension and did not require therapy. Respiratory adverse events occurred in 1 patient (3.4%) in the D–R group, and 8 patients (26.7%) in the P–R group (difference, 23.3%; 95% CI 6.2 to 40.5%; *P* = 0.026). All adverse respiratory events in the two groups were mild in severity; the insertion of asopharyngeal/ oropharyngeal airway was not required in either group.

**Table 3:** The incidence of intraoperative adverse events in both groups

	D–R group (n = 29)	P–R group (n = 30)	difference (95% CI)	*P* value
Respiratory adverse events, n (%)	1 (3.4%)	8 (26.7%)	23.3% (6.2 to 40.5%)	0.026
Cardiovascular adverse events, n (%)	6 (20.7%)	4 (13.3%)	7.4% (− 11.7 to 26.5%)	0.506
Hypertension, n (%)	2 (6.9%)	0 (0%)	6.9% (− 2.3 to 16.1%)	0.237
Hypotension, n (%)	2 (6.9%)	3 (10%)	3.1% (− 11.1 to 17.3%)	1.000
Bradycardia, n (%)	2 (6.9%)	1 (3.3%)	3.6% (− 7.6 to 14.8%)	0.612

D–R group: dexmedetomidine–remifentanil group; P–R group: propofol–remifentanil group

Table 4 shows the outcome of the arterial blood gas analysis in both groups. Partial pressure of carbon dioxide (PaCO_2_) values was significantly higher (41.52 ± 4.22 mmHg vs 44.70 ± 5.31 mmHg; difference, − 3.18 mmHg; 95% CI − 5.68 to − 0.69; *P* = 0.013). The incidence of hypercapnia was significantly lower in the D–R group (4 [13.8%]) than in the P–R group (13 [43.3%]; difference, 29.5%; 95% CI 7.8 to 51.2%; *P* = 0.012).

**Table 4:** The outcome of the arterial blood gas analysis in both groups

	D–R group (n = 29)	P–R group (n = 30)	Difference (95% CI)	*P* value
Baseline				
pH	7.42 ± 0.02	7.42 ± 0.03	− 0.002 (− 0.013 to 0.009)	0.722
PaO2 (mmHg)	79.2 ± 4.43	80.33 ± 6.11	− 1.12 (− 3.92 to 1.66)	0.422
PaCO2 (mmHg)	35.8 ± 3.30	35.70 ± 4.24	0.13 (− 1.86 to 2.11)	0.898
At the end of the procedure				
PH	7.36 ± 0.02	7.34 ± 0.02	0.017 (0.006 to 0.029)	0.003
PaO2 (mmHg)	130.03 ± 4.99	125.70 ± 7.01	4.33 (1.15 to 7.52)	0.008
PaCO2 (mmHg)	41.52 ± 4.22	44.70 ± 5.31	− 3.18 (− 5.68 to − 0.69)	0.013
Hypercapnia, n (%)	4 (13.8%)	13 (43.3%)	29.5% (7.8 to 51.2%)	0.012

D–R group: dexmedetomidine–remifentanil group; P–R group: propofol–remifentanil group; PH: pondus hydrogenii; PaO_2_: partial pressure of oxygen; PaCO_2_: partial pressure of carbon dioxide

## Discussion

This study has certain clinical implications for the sedation protocol of percutaneous ASD closure under TTE guidance. Patient tolerance is important for successful completion of a safe procedure. The technique that sedatives combining an opioid is necessary to alleviating stimulation of local anesthetic injection and reduce discomfort of the operative manipulation and the continuous probe pressed when we need to obtain good images. However, the use of sedatives combine with analgesics is usually causing intraoperative hemodynamics or respiratory complications without airway manipulation [18, 19]. Therefore, it is necessary to find an appropriate sedative protocol to improve patient comfort during the operation and reduce sedative-related adverse events.

Combination of remifentanil was administered as an analgesic requirement for the insufficient local anesthetic infiltration to alleviate spontaneous movements during painful manipulation of the delivery sheath. Moreover, considering that patient body movement under sedation is due to pain, simply deepening the level of sedation may not be the solution [18]. Therefore, we believed that continuous infusion of remifentanil enabled good tolerance of patient to this procedure and avoided the use of excessive sedatives. Although the infusion rates of remifentanil and the procedure time were similar in both groups, the time of remifentanil infusion was longer in the D–R group than in the P–R group because of the longer induction time. Remifentanil is an ultra-short-acting opioid with rapid distribution and elimination processes, and the blood concentration typically decreases by 50% 3–6 min after cessation of continuous infusion, regardless of its duration [13]. In our study, the infusion time of remifentanil would not prolong the recovery time as no difference of the value between the two groups was observed.

The main safety problem with sedation in patients is respiratory adverse events, which are mainly related to the use of sedatives and analgesics [18]. Many studies have reported that dexmedetomidine alone may not cause a decrease in respiratory or hypoventilation rates due to the central effect on respiration, but must be vigilant when combined with remifentanil [12, 13, 18]. Remifentanil is generally associated with respiratory depression, which may result in airway obstruction due to relaxation of the pharyngeal muscles. Our study found that the incidence of adverse respiratory events in the D–R group was lower than that in the P–R group. Then, we measured the PaCO_2_ level through arterial blood gas analysis, and an important finding in this study was the lower incidence of hypercapnia in the D–R group compared with the P–R group. Thus, the study further confirmed that dexmedetomidine in combination with remifentanil did not increase the risk of opioid-related respiratory depression, which similar to the results of previous study [20, 21].

Given the predictable effect in cardiovascular system, we hypothesized that there were more obvious hemodynamic changes in the dexmedetomidine–remifentanil protocol than the propofol–remifentanil protocol. Some studies have found that a dose-dependent decrease in heart rate is the most common cardiovascular effect in patients receiving dexmedetomidine, while the rate is generally not lower than 50 beats/minute, and usually, anticholinergic drugs are not required to increase the heart rate [22, 23]. Recent studies have suggested that maintaining intraoperative MABP ≥ 65 mmHg has equal clinical implications as conventional maintenance within 20% of preoperative baseline values [22, 24]. In addition, we believe that due to the fear and anxiety of the impending procedure, preoperative blood pressure may have higher initial levels before the use of sedatives. Once sedation begins, the blood pressure is probably below 20% of the baseline value during the procedure, which makes it inaccurate for assessment of hemodynamic safety. Based on these theories, we defined hypotension as an MABP < 65 mmHg to assess hemodynamic instability in this study. Rather than large or rapid bolus injection during a short period of time, continuous infusion of dexmedetomidine for an initial loading dose was helpful to prevent the hemodynamic changes that are usually associated with dexmedetomidine and to maintain stable anesthesia [25]. Finally, there was an infrequent and comparable incidence of cardiovascular adverse events in both groups, which differed from the initial hypothesis of hemodynamic changes in the dexmedetomidine–remifentanil protocol.

The primary efficacy end-point was the percentage of patients not requiring rescue sedatives based on achieving targeted sedation depth. Our study has already demonstrated that sedation protocols of both dexmedetomidine–remifentanil and propofol–remifentanil combinations have comparable efficacy for providing sufficient sedative and analgesic effects. However, physicians favored the propofol–remifentanil-based sedation protocol perhaps due to the shorter induction time. Although the incidence of cardiovascular adverse events was comparable and such events were easily managed in both groups, the dexmedetomidine–remifentanil protocol had superior airway security since it is associated with fewer hypercapnia and respiratory adverse events than the propofol–remifentanil protocol. The best approach to sedation for patients in percutaneous closure of ASD is to choose a sedation regimen tailored according to the clinical risk assessment. For those patients with the uncertainties in airway safety, dexmedetomidine–remifentanil-based sedation protocol may be a suitable sedation approach.

Intracardiac shunts can change the onset time of sedation in ASD patients [25]. Previous study demonstrated that significantly delayed pharmacodynamics responses to neuromuscular blocking agent cisatracurium were observed in patients with septal defects [26]. In this study, there was a longer induction time to achieve targeted levels of sedation with dexmedetomidine than with propofol, which was also similar to the results of previous research above the patients without intracardiac shunts [12]. However, there is no clinical study involving adequate number of patients has been done to explore the magnitude of difference in the induction time of anesthesia in patients with or without intracardiac shunt [27, 28]. Simple dosage regimens based only on patient weight may not result in stable effect-site concentrations because of the complex pharmacokinetics and pharmacodynamics of each drug. Further exploration of pharmacokinetics and pharmacodynamics of sedatives in patients with intracardiac shunts assumed great significance in the formulation of a population-specific dosage regimen to achieve optimized therapy.

## Conclusions

In the term of efficacy, both sedation protocols have comparable efficacy for providing sufficient sedative and analgesic effects, however, the time to target sedation level was more rapid and the surgeon satisfaction score was higher in the propofol–remifentanil protocol than in the dexmedetomidine–remifentanil sedation protocol. In terms of sedation safety, considering that the hemodynamic stability was comparable, we concluded that dexmedetomidine–remifentanil protocol had superior airway security since it was associated with fewer hypercapnia and respiratory adverse events.

## Data Availability

Data used for this study are available upon request. All relevant data are within the manuscript and its Supporting Information files.

## Abbreviations

ASD: Atrial septal defects
TTE: Transthoracic echocardiography
TEE: Transesophageal echocardiography
D–R group: Dexmedetomidine–remifentanil group
P–R group: Propofol–remifentanil group
ASA: American Society of Anesthesiologists
OAA/S: Observer’s Assessment of Alertness/Sedation
BIS: Bispectral index score
VAS: Visual analog scale
PH: Pondus hydrogenii
PaO2: Partial pressure of oxygen
PaCO2: Partial pressure of carbon dioxide
